# Homeobox B2 promotes malignant behavior and contributes to the radioresistance of nasopharyngeal carcinoma by regulating forkhead box protein O1

**DOI:** 10.7150/ijms.93128

**Published:** 2024-03-17

**Authors:** Jinhai Chen, Min Mao, Zhaoen Ma, Jie Liu, Minqiong Jiang, Guangui Chen, Yali Xu

**Affiliations:** 1 Department of Otolaryngology, The Second Affiliated Hospital of Guangzhou Medical University, Guangzhou, China; 2 Department of Anesthesiology, The Second Affiliated Hospital of Guangzhou University of Chinese Medicine, Guangzhou, China; 3 Department of Nursing, The Second Affiliated Hospital of Guangzhou Medical University, Guangzhou, China

**Keywords:** nasopharyngeal carcinoma, radioresistance, apoptosis, HOXB2, FOXO1

## Abstract

**Background:** Nasopharyngeal carcinoma (NPC) is an epithelial tumor of the head and neck with heterogeneous racial and geographical distributions. Homeobox B2 (HOXB2) is a tumor promoter in many cancers. However, the biological role of HOXB2 in NPC has not been elucidated.

**Methods:** Bioinformatics analysis was performed to identify the differentially expressed genes (DEGs) between samples of patients with radiosensitive and radioresistant NPC. qRT-PCR, western blotting and immunohistochemistry were used to detect the expression levels of the corresponding mRNA and proteins. Cell viability was detected by CCK-8 assay and colony-forming capability was evaluated using colony formation assays. Further, migration and invasion abilities were examined using wound-healing and transwell chamber assays, respectively. Cellular apoptosis after irradiation was assessed using flow cytometry and terminal deoxynucleotidyl transferase dUTP nick-end labeling (TUNEL) staining.

**Results:** HOXB2 was identified as a potential regulator of radioresistance in NPC. Our in vitro results indicate that HOXB2 overexpression (HOXB2-OE) promoted malignant behaviors including invasion, migration, proliferation, and inhibited the irradiation-induced apoptosis of NPC cells. Consistent with these results, HOXB2 knockdown (HOXB2-sh) exhibited the opposite trends in these biological activities. Kyoto Encyclopedia of Genes and Genomes (KEGG) analysis showed that the DEGs were enriched in the FOXO signaling pathway. Mechanistically, western blotting showed that HOXB2-OE inhibited forkhead box protein O1 (FOXO1) expression in NPC cells. Thereafter, we transferred the FOXO1-OE plasmid to HOXB2-OE NPC cells and found that overexpression of FOXO1 reversed cell proliferation, migration, invasion, and radioresistance profiles promoted by HOXB2 overexpression.

**Conclusion:** Our findings showed that HOXB2 acts as a tumor promoter in NPC, activating malignant behaviors and radioresistance of tumors via FOXO1 regulation. Moreover, the inactivation of HOXB2 or activation of FOXO1 are potential strategies to inhibit tumor progression and overcome radioresistance in NPC.

## Introduction

Nasopharyngeal carcinoma (NPC) is a rare malignant cancer arising from the nasopharyngeal mucosal lining. Exhibiting heterogeneous racial and geographical distributions, NPC is particularly common in East and South East Asia 1,2. Concurrent radio-chemotherapy has become the primary treatment for NPC in clinical practice. With the development of intensity-modulated radiotherapy and the widespread application of chemoradiotherapy, the 5-year overall survival rate of patients with non-metastatic NPC has risen to 54.8-75.7% 3. However, 10-20% of patients suffer from locoregional recurrence after primary treatment 4. Once recurrence or distance metastasis occurs, the 5-year survival of these patients declines below 50% 5. Accumulating evidence has demonstrated that radioresistance of tumor cells is the major reason for residual or recurrent/distant cancers, ultimately resulting in poor prognosis 6,7,8. Therefore, further investigation is required to elucidate the mechanism of radioresistance.

The rapid development of bioinformatics enables researchers to determine the hub genes and molecular mechanisms of cancer progression to identify new potential therapeutic targets of drugs or radiation resistance. In the present study, we screened homeobox B2 (HOXB2) as a potential regulator of the progression and radioresistance of NPC with a comprehensive bioinformatics, which its expression in patient tissues was also increased by immunohistochemistry. *HOX* genes are highly conserved homeotic genes that were first identified in the fruit fly, *Drosophila melanogaster* 9. Aberrations in *Hox* gene expression have been reported in abnormal development and malignancy, indicating that dysregulation of these genes contributes to tumor initiation and progression 10,11. *HOXB2* is a member of the B cluster of homeobox transcription factors localized on chromosomes 17 that acts as either an oncogene or tumor suppressor with tissue-specific regulation. A previous functional in vivo screen identified HOXB2 as a negative regulator of tumor growth that decreased proliferation in mammary adenocarcinoma tumors 12. This result is at odds with subsequent HOXB2 studies in Wilms tumor, glioma, colon cancer, and esophageal squamous cell carcinoma, in which overexpression was associated with malignancy 13,14,15,16. Nevertheless, the functions of HOXB2 in NPC have not been studied.

Forkhead box protein O1 (FOXO1) belongs to the forkhead box O (FOXO) family of transcription factors, which is a tumor-suppressing factor that inhibits carcinogenesis 17. However, some studies revealed a controversial role in the development of tumors. For example, Wang et al. found that FOXO1 has a tumor-promoting effect on inducing the infiltration of M2 macrophages and led to poor prognosis in esophageal squamous cell carcinoma (ESCC) patients 18. These conflicting findings suggested that the role of FOXO1 may alter based on the type of cancer tissues. In our study, KEGG analysis showed that FOXO signaling pathway could potentially contributed to radioresistance in NPC. Taken together, we aimed to elucidate the biological role of HOXB2 and the effect of the HOXB2/FOXO1 interaction on NPC progression and radioresistance. Our findings may indicate new potential therapeutic targets to overcome radioresistance and improve the prognosis of patients with NPC.

## Materials and methods

### Bioinformatics analysis

Gene expression profiles and clinical data from GSE32389 dataset were downloaded. After excluding outlier samples, eight RSNPC and twelve RRNPC samples were included in the subsequent analysis. The values of |log fold change| > 0 and *p* < 0.05 were used to identify the differentially expressed genes (DEGs) between RSNPC and RRNPC. Thereafter, Gene Ontology (GO) and Kyoto Encyclopedia of Genes and Genomes (KEGG) pathway enrichment analyses were performed using the Cytoscape 3.9 software and DAVID tool (https://david.ncifcrf.gov/), respectively. The corresponding pathways were considered significantly enriched if *p* < 0.05.

### Immunohistochemistry

Immunohistochemistry (IHC) was performed on formalin-fixed, paraffin-embedded tissue specimens from patients with chronic nasopharyngitis, complete recovery of NPC tissues, and recurrent NPC. Briefly, tissues were blocked using goat serum and incubated with primary anti-HOXB2 antibody (China, ER63708, 1:250) at 4 ° C overnight. Samples were then incubated with biotinylated secondary antibody diluted 1:1000.

### Cell lines and culture

Cells from the NPC epithelioid cell line CNE-1 and epithelial tumor cell line HONE-1 were provided by The State Key Laboratory of Oncology at Sun Yat-sen University Cancer Center. The cells were cultured in Dulbecco's modified Eagle's medium (DMEM; HyClone Laboratories, San Angelo, TX, USA) supplemented with 10% fetal bovine serum (FBS; Hyclone) and 1% penicillin-streptomycin (Beijing Solarbio Science & Technology, Beijing, China). All cells were cultured in a humidified incubator in a 5% CO2 atmosphere at 37°C.

### Lentiviral transduction of NPC cells

cDNA encoding human HOXB2 (https://www.ncbi.nlm.nih.gov/nuccore/NM_002145.4) was inserted into a P-cmv-mcs-EF1-EGFP-T2A-PURO lentiviral vector to produce a HOXB2 overexpression (HOXB2-OE) vector. The shRNA sequence 5' CCGGGCGGCCTTTAGCCGTTCGCTTACTCGAGTAAGCGAACGGCTAAAGGCCGCTTTTTG 3', which targets HOXB2 mRNA, was transferred into P-u6-MCS-EF1-EGFP-T2A-PURO lentiviral vector to create a HOXB2 knockdown (HOXB2-sh) plasmid. Each recombinant lentiviral plasmid and packaging helper plasmid (pSPXA2/pMD2.G) were co-transfected 1:3 into 293T cells with 20% serum for fluid exchange. The virus supernatant was collected 48 h later and used to infect the NPC cells. Transfected NPC cells were subjected to continuous pressure screening with puromycin for 2 weeks, and stable high-/low- expression NPC cells were obtained. cDNA encoding human FOXO1 (https://www.ncbi.nlm.nih.gov/nuccore/NM_002015.4) was inserted into a PCDNA3.1 vector for FOXO1 overexpress (FOXO1-OE).

### Quantitative real-time PCR

TRIzol (Invitrogen, Thermo Fisher Scientific, Waltham, MA, USA) was used to isolate total RNA, and the PrimeScript reverse transcription (RT)-PCR kit (Takara Bio, Shiga, Japan) was used to synthesize cDNA according to the manufacturer's instructions. Glyceraldehyde 3-phosphate dehydrogenase (*GAPDH*) was used as an internal control. All experiments were performed at least in triplicate. The primer sequences used for qRT-PCR were as follows: *HOXB2* forward, 5'-CGCCAGGATTCACCTTTCCTT-3' and reverse, 5'-CCCTGTAGGCTAGGGGAGAG-3'; *GAPDH* forward, 5'-AGAAGGCTGGGGCTCATTTG-3' and reverse, 5'-AGGGGCCATCCACAGTCTTC-3'.

### Cell-counting Kit-8 assay

At 24-h intervals (0, 24, 48, and 72 h), 10 μl of the Cell-Counting Kit-8 (CCK-8) reagent (Beyotime Biotechnology, Shanghai, China) was added to each well and the cells were incubated for 1 h. The optical density was measured at 450 nm using a microplate reader (Thermo Fisher Scientific).

### Colony formation assay

Stably transfected cells were seed in 6-well plates for a week in the media contain 10% FBS. Subsequently, the colonies were fixed with 4% formaldehyde for 30 min, stained with 1% crystal violet for 15 min, photographed, and counted under a light microscope (Olympus Life Science, Tokyo, Japan).

### Western blotting

Whole cell lysate was extracted using radioimmunoprecipitation assay buffer (Beyotime Biotechnology) containing protease inhibitor (Roche, Indianapolis, IN, USA). Proteins were electrophoresed on 10% sodium dodecyl sulfate polyacrylamide gels and transferred to polyvinylidene difluoride (PVDF) membranes (MilliporeSigma, Burlington, MA, USA) following the manufacturer's instructions. The PVDF membrane was blocked with 5% milk in Tris-buffered saline and 0.1% Tween for 1 h at room temperature and incubated overnight at 4 °C with the relevant antibodies (HOXB2, FOXO1, and GAPDH; Cell Signaling Technology, Danvers, MA, USA). The membranes were rinsed and incubated for 1 h with the corresponding peroxidase-conjugated secondary antibodies (Abcam, Waltham, MA, USA). Chemiluminescent detection was performed using an enhanced chemiluminescence kit (Thermo Fisher Scientific). Membranes were visualized by using the ChemiDoc Imaging System (Bio-Rad, Hercules, CA, USA).

### Migration and invasion assays

Briefly, cells were seeded in upper transwell chambers in a Roswell Park Memorial Institute (RPMI)-1640 medium containing 10% FBS. After culturing for 24 h, cells that invaded the lower transwell chamber were fixed with 4% paraformaldehyde and stained with 0.1% crystal violet. The number of cells in five fields in each well was counted.

Cells were then seeded into 6-well plates at a density of 1 × 10^6^ cells per well in a RPMI-1640 medium supplemented with 10% FBS. After the cells reached a confluence of 80%, the cultured monolayers were mechanically scraped with 200-μl pipette tips and cultured in the RPMI-1640 medium containing 0.5% FBS for 48 h. Images were captured at 0, 24 and 48 h.

### Cell apoptosis

The cells were irradiated with 3-Gy X-ray, harvested after culture for 24 h, and stained with annexin V/propidium iodide for 15 min. Apoptosis was analyzed by flow cytometry (ACEA Biosciences, San Diego, CA, USA). The terminal deoxynucleotidyl transferase dUTP nick-end labeling (TUNEL) reaction mixture was placed in a dark, humid environment at 37 °C for 1 h. Finally, 4′,6-diamidino-2-phenylindole (DAPI) was used to simultaneously stain the nuclei. TUNEL-positive NPC cells were analyzed under a fluorescence microscope.

### Statistical analyses

Statistical analyses were conducted using SPSS 20.0 (IBM, Armonk, NY, USA) and GraphPad Prism software (version 8.0.1; GraphPad, San Diego, CA, USA). Values are presented as the mean ± standard deviation from at least three independently performed experiments. The measurement data were analyzed by one-way ANOVA or Student's t-test, *p* < 0.05 was considered to be statistically significant.

## Results

### Experimental validation of bioinformatic data

**Figure 1:**
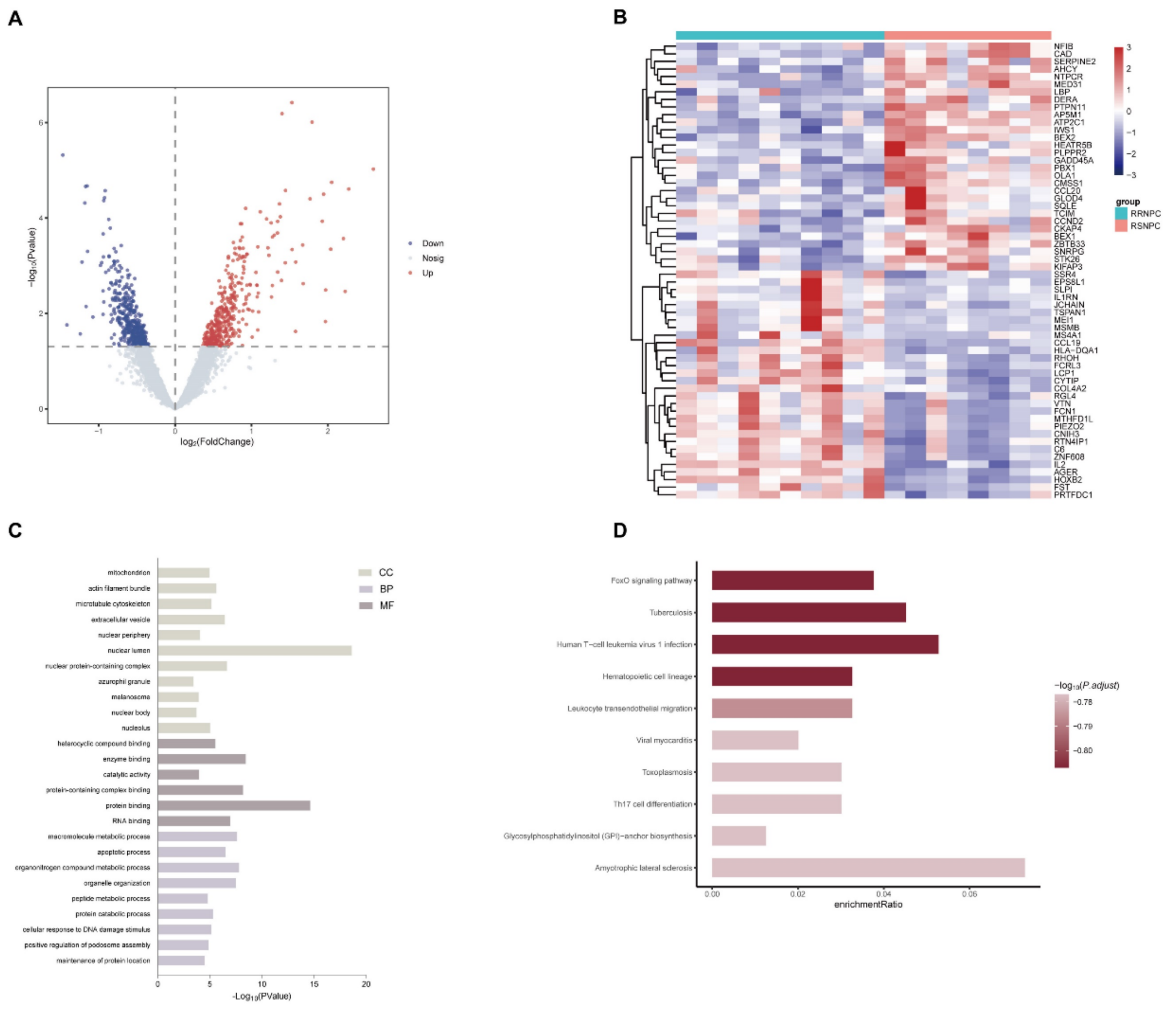
** Bioinformatic identification of DEGs and pathways associated with NPC radioresistance.** (A) Volcano plots were used to visualize the DEGs between radiosensitive and radioresistance NPC samples. (B) Heatmap of the top 30 significantly up- and downregulated DEGs. (C) GO analysis of the categories of biological process (BP), cellular component (CC) and molecular function (MF) of the DEGs. (D) KEGG analysis of the enriched pathways of DEGs. DEG, differentially expressed gene; GO, Gene Ontology; KEGG, Kyoto Encyclopedia of Genes and Genomes; NPC, nasopharyngeal carcinoma.

We firstly analyzed the microarray information of the dataset GSE32389 acquired from the GEO database and identified 395 upregulated and 425 downregulated DEGs between RSNPC and RRNPC samples (Fig. 1A). The top 30 up- and downregulated DEGs were presented in a heatmap (Fig. 1B). To determine the potential signaling pathway regulating the radio-responsiveness of NPC, we performed GO (Fig. 1C) and KEGG (Fig. 1D) analyses of the DEGs. The GO results indicated that the significantly enriched terms were mainly involved in nuclear lumen, protein binding and organonitrogen compound metabolic process. Furthermore, KEGG enrichment analysis showed that signaling pathways potentially contributed to radioresistance in NPC, including the FOXO signaling pathway, tuberculosis, and Human T-cell leukemia virus 1 infection.

**Figure 2:**
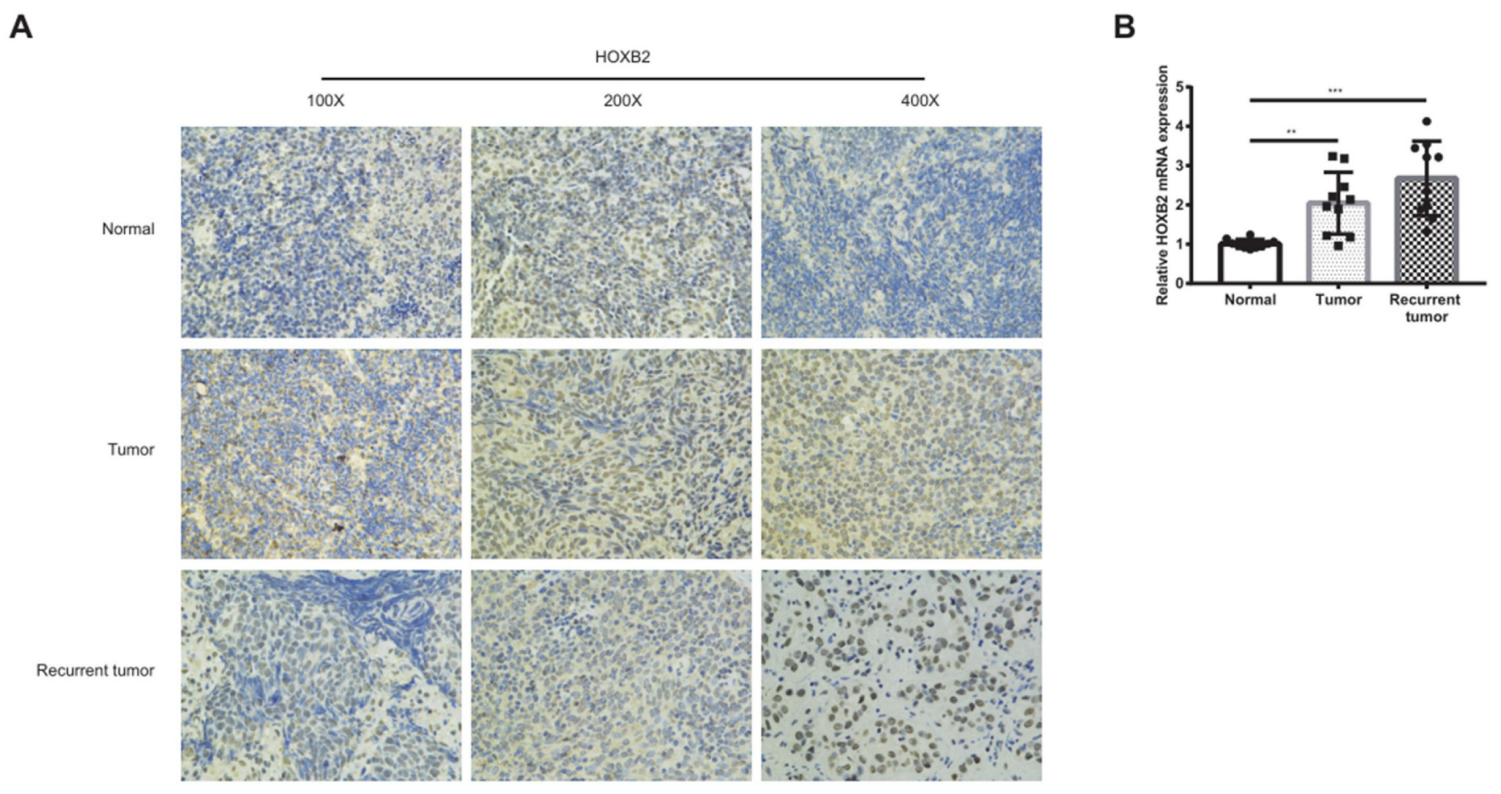
** Immunohistochemical analysis and qPCR to detect the expression of HOXB2 in clinical samples.** (A) Immunohistochemical analysis to detect the expression of HOXB2 in clinical samples. (B) qPCR to quantify the expression of HOXB2 in clinical samples. Tumor, patient with complete recovery from NPC; Recurrent tumor, patient with recurrent NPC. HOXB2, homeobox B2.

As shown in Figure 1A, HOXB2 was 3.5 times more expressed in the RRNPC than RSNPC samples. We verified the expression pf HOXB2 in samples from patients with NPC using IHC. Representative IHC images showed that HOXB2 protein levels were higher in NPC tissues than in chronic nasopharyngitis (Fig. 2A).

Taken together, HOXB2 could be considered as potential promoter gene in regulating NPC progression and radioreistance.

### Effects of HOXB2 on viability and motility in NPC cell lines

**Figure 3:**
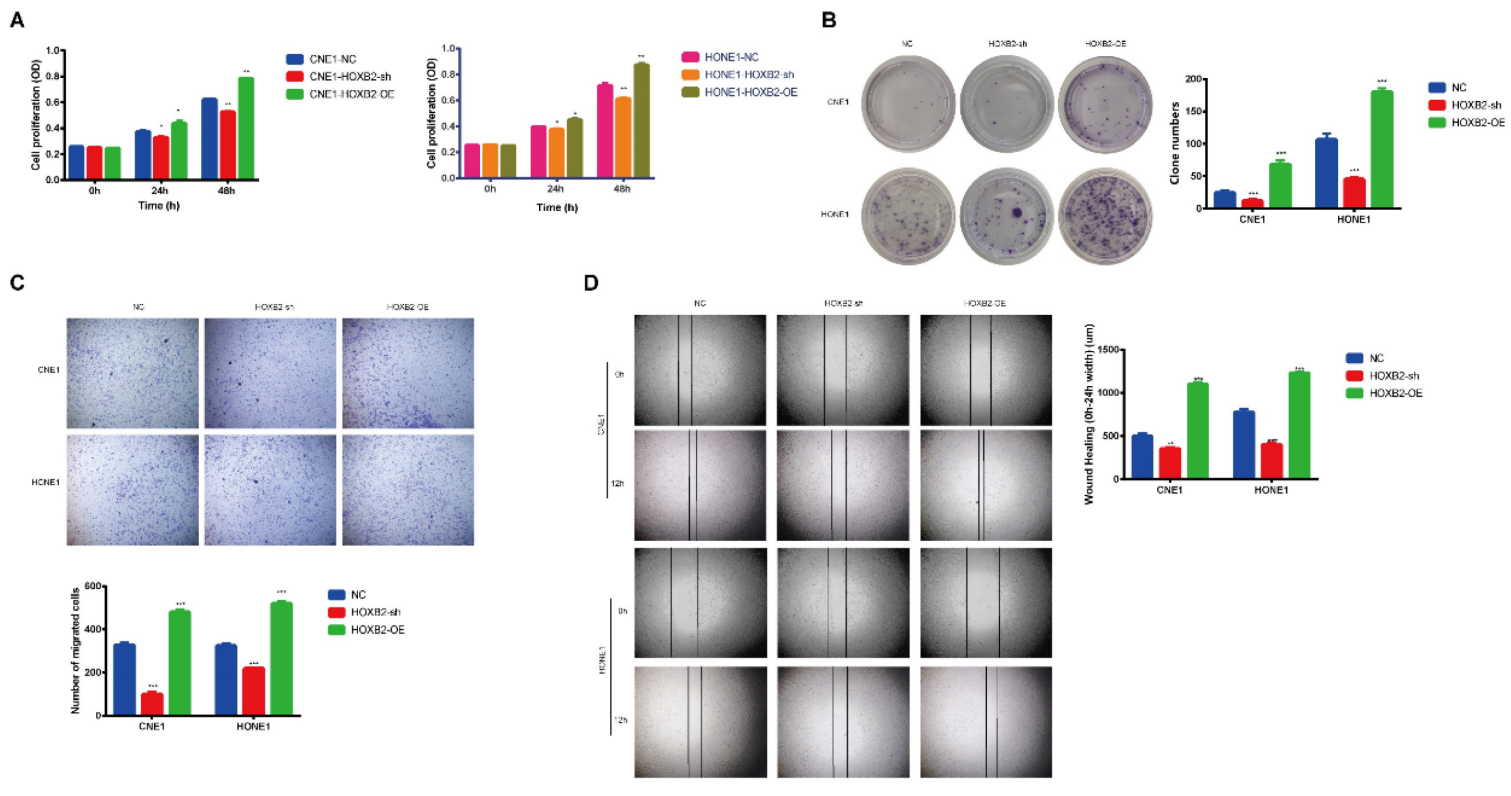
** The effects of HOXB2 on viability and motility of NPC cell lines.** (A) CCK-8 assays were utilized to detect the proliferation abilities in NPC cell lines after 24h and 48h. (B) Colony-forming ability of NPC cells. (C) Representative images of transwell assays and quantitative assessment of the number of cells invading the lower chamber. (D) Wound-healing assays were utilized to test wound closure in NPC cells. The wound space was photographed at 0 and 12h. CCK-8, Cell-Counting Kit-8; HOXB2-sh, homeobox B2 knockdown; HOXB2-OE, homeobox B2 overexpression; NC, negative control; NPC, nasopharyngeal carcinoma; TUNEL, terminal deoxynucleotidyl transferase dUTP nick-end labeling.

To illustrated the biological functions of HOXB2 in NPC, CNE1 and HONE1 cells stably overexpressed (HOXB2-OE) and knocked down HOXB2 (HOXB2-sh) were established. The CCK-8 assay results (Fig. 3A) showed that HOXB2-OE markedly promoted NPC cell proliferation, whereas HOXB2 inhibition significantly attenuated cell viability compared with that of the negative control (NC) cells after 24h and 48h (*p* < 0.05). These findings were consistent with the colony formation results (Fig. 3B), which indicated that the number of colonies of HOXB2-OE NPC cells were significantly increased (*p* < 0.05). In contrast, the number of colonies of HOXB2-sh NPC cells was significantly reduced compared with that of the NC cells (*p* < 0.05).

The impact of HOXB2 on cell migration and invasion was detected using transwell and wound healing assays (Fig. 3C, D). HOXB2-OE significantly enhanced cell migration and invasion, whereas knockdown of HOXB2 resulted in all NPC cell lines displaying reduced invasion and migration abilities.

Collectively, these results indicated that HOXB2 stimulates cell viability, colony formation, migration, and invasion in NPC cells.

### HOXB2 was associated with radiation-induced apoptosis in NPC cells

**Figure 4:**
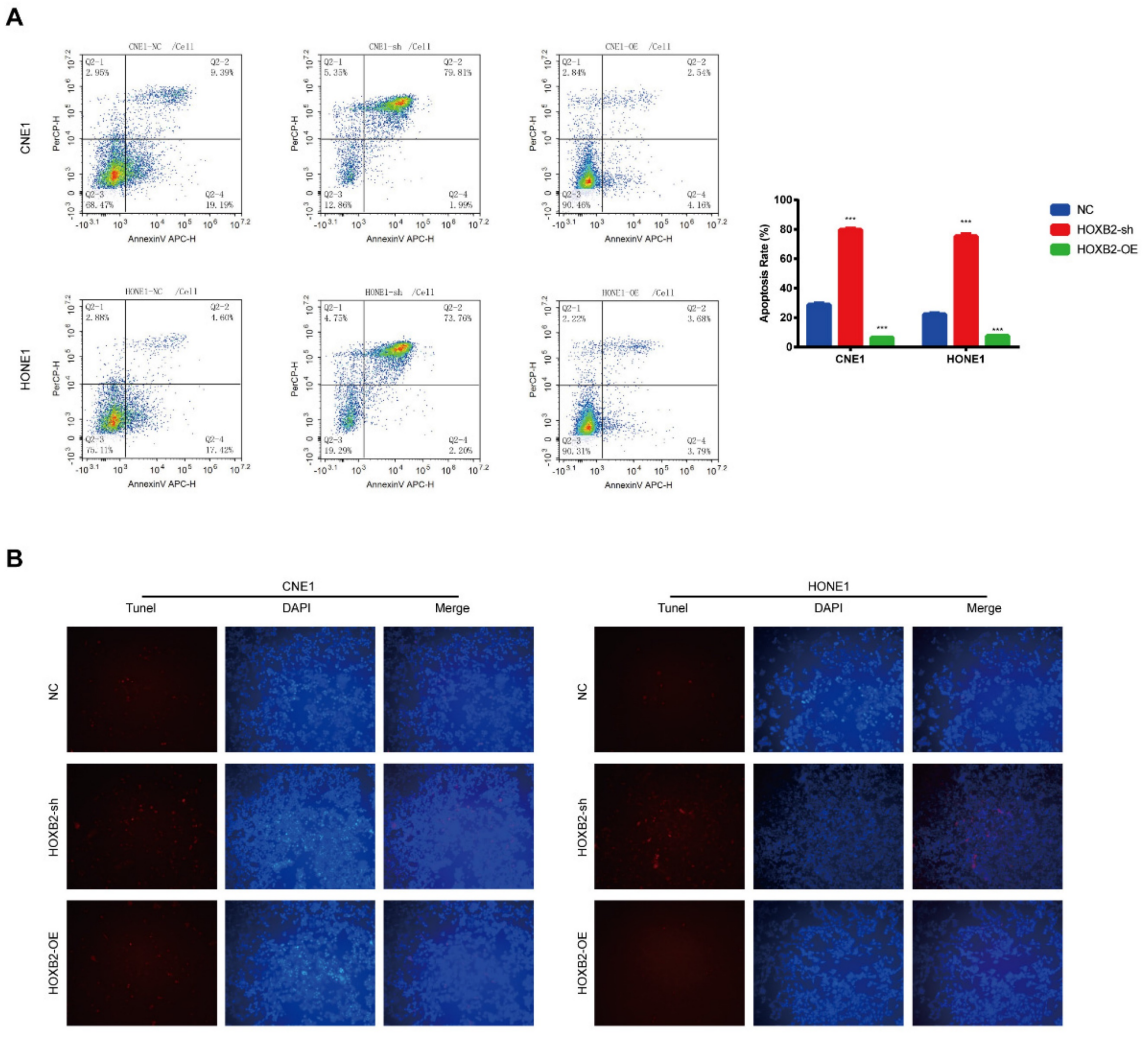
** Radiation-induced apoptosis of NPC cells.** (A) Flow cytometry was performed to detect the apoptosis rates of NPC cells after transfection. (B) The TUNEL assay showed changes in apoptosis in NPC cell lines after transfection. HOXB2-sh, homeobox B2 knockdown; HOXB2-OE, homeobox B2 overexpression; NC, negative control; NPC, nasopharyngeal carcinoma; TUNEL, terminal deoxynucleotidyl transferase dUTP nick-end labeling.

Apoptosis is a critical cellular response to radiotherapy. To illustrate the role of HOXB2 in regulating radioresistance in NPC, flow cytometry and TUNEL assays were performed to detect apoptosis induced by irradiation with 3 Gy after 24 h. In the CNE-1 cells, the apoptosis rates of the NC, HOXB2-sh, and HOXB2-OE groups after receiving irradiation treatment were 28.58%, 81.8% and 6.7%, respectively (*p* < 0.001). Similar results were observed in the HONE-1 groups (*p* < 0.001) (Fig. 4A). Moreover, we found that the number of TUNEL-positive NPC cells was higher in the HOXB2-sh group than in the NC group, whereas the opposite trend was observed in HOXB2-OE group (Fig. 4B).

In summary, HOXB2 suppressed radiation-induced apoptosis and consequently promoted radioresistance in NPC cells.

### FOXO1 overexpression reversed viability and motility functions in HOXB2-OE NPC cell lines

**Figure 5:**
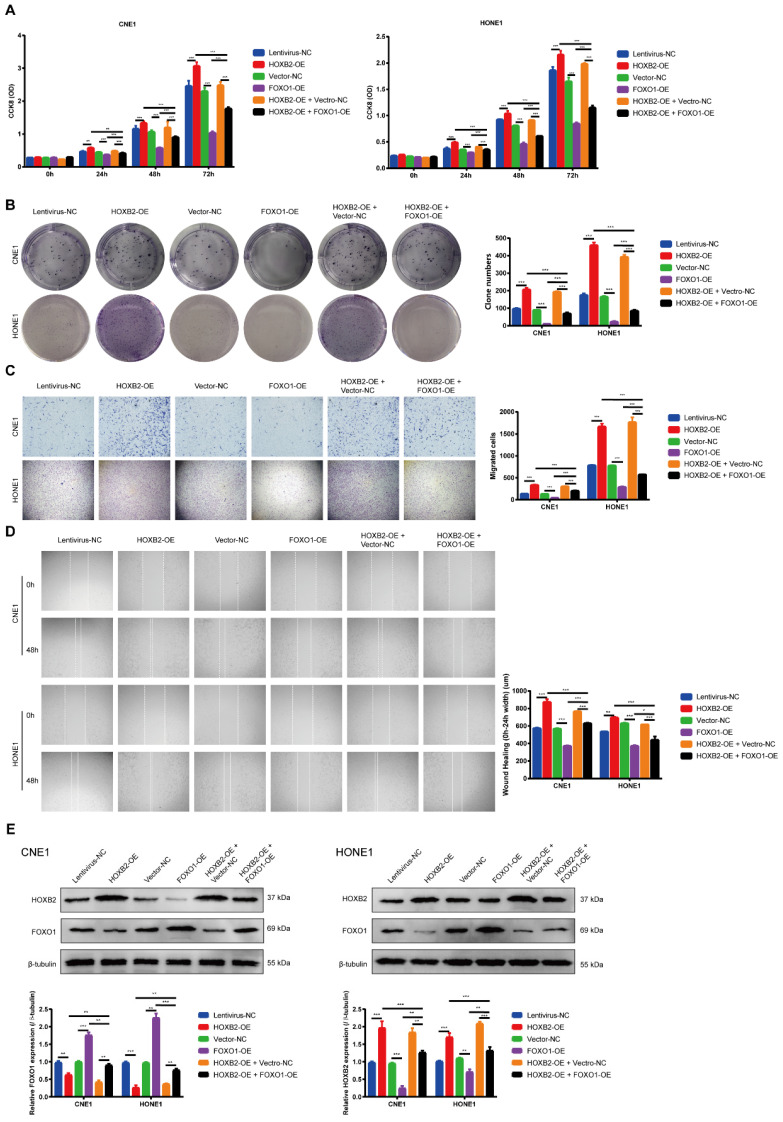
** FOXO1 overexpression reduced viability and motility of NPC cells and reversed viability and motility of HOXB2-OE NPC cells.** (A) CCK-8 assays were utilized to detect the proliferation abilities of NPC cells after 24 h, 48 h and 72 h. (B) Colony-forming ability of NPC cells. (C) Representative images of transwell assays and quantitative assessment of the number of cells invading the lower chamber. (D) Wound-healing assays were utilized to test wound closure in NPC cells. The wound space was photographed at 0 and 48h. (E) Western blotting was performed to detect the expression of FOXO1 and HOXB2 in transfected NPC cells. CCK-8, Cell-Counting Kit-8; FOXO1-OE, forkhead box protein O1 overexpression; HOXB2-OE, homoeobox 2 overexpression; HOXB2-OE + FOXO1-OE, co-transfection with HOXB2-OE and FOXO1-OE in NPC cell lines; NC, negative control; NPC, nasopharyngeal carcinoma.

To ascertain whether the FOXO signaling pathway is involved in regulating NPC progression, we performed western blotting analysis to determine the FOXO1 expression after HOXB2-OE transfection in NPC cells. These results indicated that HOXB2-OE inhibited FOXO1 expression in NPC cells (Fig. 5E). Similarly, the CCK8 results showed that the proliferation ability of FOXO1-OE NPC cells was significantly lower than that of vector-NC cells (*p* < 0.001, Fig. 5A). The number of FOXO1-OE NPC cell colonies was also significantly decreased (*p* < 0.001, Fig. 5B). Furthermore, transwell wound-healing assays indicated that FOXO1-OE led to diminished invasion and migration abilities of all NPC cell lines (*p* < 0.001, Fig. 5C, D).

To verified this mechanism, we co-transfected NPC cells with FOXO1-OE and HOXB2-OE. The rescue experiments confirmed that overexpression of FOXO1 abolished the HOXB2-induced stimulation of proliferation and migration in NPC (*p* < 0.001) (Fig. 5).

Collectively, these results indicated that HOXB2 promoted NPC progression by regulating FOXO1 expression.

### FOXO1 overexpression suppressed HOXB2-mediated radioresistance by influencing apoptosis after irradiation

**Figure 6:**
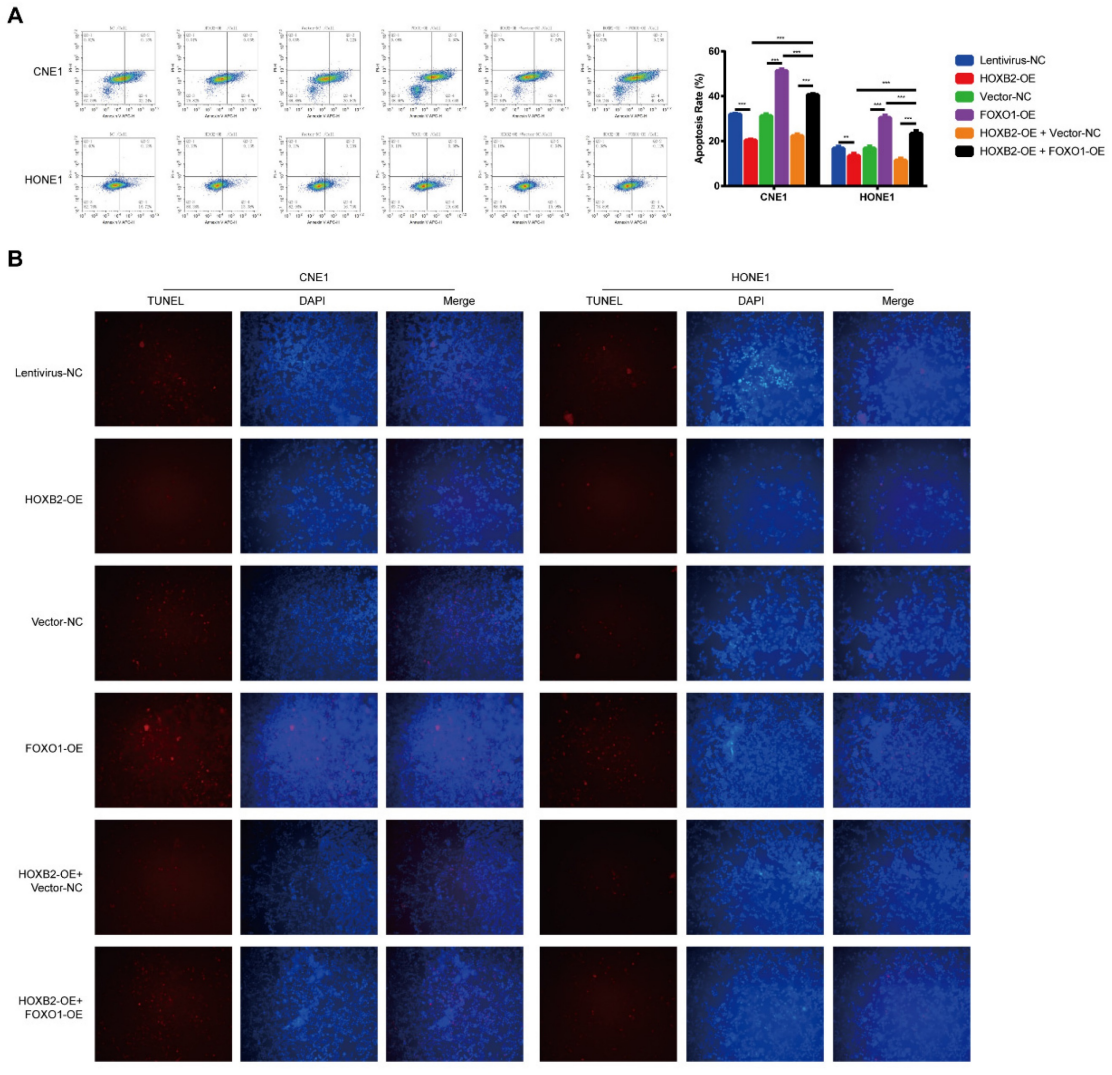
** FOXO1 overexpression promoted radiation-induced apoptosis in NPC cell lines and revered radiation-induced apoptosis in HOXB2-OE NPC cell lines.** (A) Flow cytometry was performed to detect the apoptosis rates of NPC cells after transfection. (B) The TUNEL assay showed changes in apoptosis in NPC cell lines after transfection. FOXO1-OE, forkhead box protein O1 overexpression; HOXB2-OE, homeobox 2 overexpression; HOXB2-OE + FOXO1, co-transfection with HOXB2-OE and FOXO1-OE in NPC cell lines; NC, negative control; NPC, nasopharyngeal carcinoma; TUNEL, terminal deoxynucleotidyl transferase dUTP nick-end labeling.

To verify the effect of the HOXB2/FOXO1 interaction on NPC cell radioresistance, we further explored whether FOXO1 overexpression could reverse radiation-induced apoptosis. In CNE1 group, FOXO1-OE cells presented higher apoptosis rates than vector-NC cells (51.26% vs. 31.06%, *p* < 0.001). The same trend was observed in the HONE-1 group, suggesting that FOXO1 enhances NPC radiosensitivity. Notably, FOXO1 overexpression attenuated the effects of HOXB2 in irradiated CNE-1 and HONE-1 cells. Flow cytometry results showed increased apoptosis of HOXB2-OE + FOXO1-OE co-transfected cells compared with HOXB2-OE + vector-NC cells (*p* < 0.001) (Fig. 6A). Similarly, the number of TUNEL-positive cells was higher in the HOXB2-OE + FOXO1-OE group than in the HOXB2-OE + vector-NC group (Fig. 6B).

These results indicated that NPC radioresistance was synergistically regulated by HOXB2 and FOXO1.

## Discussion

Radiotherapy remains one of the most widely used and effective clinical methods for treating tumors. However, intrinsic or acquired radioresistance of tumor cells leads to disease recurrence and poor survival rates 19,20. Therefore, identifying targets that improve radiosensitivity may be a promising strategy against NPC. Based on microarray data analysis from the GEO database and HOXB2 expression patterns in the clinical experimental validation, we believe that HOXB2 is a potential target for regulating the malignant behavior and radioresistance of NPC. Recent studies suggest that HOXB2 participates in tumor development and progression 21. For example, Pan et al. demonstrated that lncRNA RGMB-antisense RNA I promote glioma progression by increasing HOXB2 expression 22. Further, Li et al. reported that circ-0001785 regulates osteosarcoma pathogenesis by upregulating HOXB2 expression 16. Another study by Yang et al. revealed that HOXB2 promoted the proliferation and invasion of colon cancer cells by increasing the expression of CCT6A in colon cancer cells 23. Currently, there is a lack of literature report on the relationship between HOXB2 and nasopharyngeal carcinoma, as well as its correlation with HOXB2 and radiotherapy response. Our results showed that HOXB2-OE enhanced, while HOXB2-sh reduced the viability and motility of NPC cells in vitro. Numerous studies have demonstrated that the inhibition of apoptosis is closely related to tumor cell radioresistance 24. To further elucidate the role of HOXB2 in NPC cell radiosensitivity, we performed flow cytometry and TUNEL assays to detect irradiation-induced apoptosis. HOXB2-sh predisposed NPC cells to apoptosis, whereas HOXB2-OE had the opposite effect. These results demonstrated that HOXB2 not only exhibits carcinogenic effects in NPC, but also enhances radioresistance in NPC cell lines.

Multiple signaling pathways are involved in radiotherapy resistance of NPC including Wnt/β-catenin, NF-κB, Notch, AKT, etc. 25 However, the mechanism of NPC radioresistance remains elusive. KEGG analysis demonstrated that FOXO may be a potential pathway for regulating NPC radioresistance. Yang et al. performed a KEGG analysis of the radioresistance of differentially expressed circRNA target miRNAs and showed that the FOXO signaling pathway is involved in circRNA function 26. Guo et al. analyzed DEGs and microRNA from GEO and identified *JUN* as a core hub gene in the regulation of NPC radioresistance by the nucleotide oligomerization domain (NOD)-like receptor signaling pathway, interleukin-17 signaling pathway, transforming growth factor beta signaling pathway, and FOXO signaling pathway 27. Therefore, we sought to determine whether HOXB2 regulates NPC progression and radioresistance via the FOXO signaling pathway. The transcription factor FOXO1 acts as a tumor suppressor, inhibiting the proliferation, migration, and invasion of various cancers, such as hepatocellular, lung, prostate, and colon cancer 17,28,29. By establishing FOXO1-OE NPC cells, we observed that suppressed proliferation, colony formation, migration, and invasion in comparison with vector-NC cells, supporting the anti-neoplastic action of FOXO1. Moreover, Wang et al. found that the activation of FOXO1 may be a strategy to overcome radioresistance in bladder cancer 30. Similarly, Huang et al. observed that human glioma cell radiosensitivity was enhanced by lipopolysaccharide-induced tumor necrosis factor alpha (LITAF) via the FOXO1 pathway 31. In the NPC field, Li et al. demonstrated that FOXO1 functions as a tumor suppressor to prevent NPC pathogenesis. The authors observed that FOXO1 not only controlled tumor stemness and metastasis, but also sensitized NPC cells to cisplatin in vitro and in vivo 32.

Deng et al. found that miR-613 enhances the radiosensitivity of NPC cells by inhibiting the downstream signal transducer and activator of transcription 1 (STAT1)/FOXO1 pathway 33. In our study, western blotting results showed that FOXO1 levels were markedly decreased in NPC cells after HOXB2 overexpression. Overexpression of FOXO1 promoted radiation-induced apoptosis in NPC cells, supporting the role of FOXO1 in radioresistance. We co-transfected NPC cells with HOXB2-OE and FOXO1-OE to assess their joint activity. As expected, the effects of HOXB2 on NPC progression and radioresistance were reversed by the FOXO1-OE vector. Taken together, our findings highlight that NPC progression and radioresistance are synergistically regulated by HOXB2 and FOXO1.

However, this study had several limitations. First, we did not confirm the binding relationship between HOXB2 and FOXO1. Second, we only conducted functional experiments and relevant mechanistic studies in NPC cell lines. Therefore, our findings warrant in vivo validation experiments in future studies.

In conclusion, our study provides the first preliminary evidence that HOXB2 acts as a tumor promoter in NPC progression and enhances radioresistance by regulating FOXO1 expression. Consequently, the HOXB2 inactivation or FOXO1 activation may be potential strategies to inhibit tumor progression and overcome radioresistance in NPC.

## Supplementary Material

Supplementary figure.

## References

[1] Chen YP, Chan ATC, Le QT (2019). Nasopharyngeal carcinoma. Lancet.

[2] Bossi P, Chan AT, Licitra L (2021). Nasopharyngeal carcinoma: ESMO-EURACAN Clinical Practice Guidelines for diagnosis, treatment and follow-up†. Ann Oncol.

[3] Blanchard P, Lee A, Marguet S (2015). Chemotherapy and radiotherapy in nasopharyngeal carcinoma: an update of the MAC-NPC meta-analysis. Lancet Oncol.

[4] Lee AWM, Ng WT, Chan JYW (2019). Management of locally recurrent nasopharyngeal carcinoma. Cancer Treat Rev.

[5] Wu F, Wang R, Lu H (2014). Concurrent chemoradiotherapy in locoregionally advanced nasopharyngeal carcinoma: treatment outcomes of a prospective, multicentric clinical study. Radiother Oncol.

[6] Sun X, He Z, Guo L (2021). ALG3 contributes to stemness and radioresistance through regulating glycosylation of TGF-β receptor II in breast cancer. J Exp Clin Cancer Res.

[7] Bai X, Ni J, Beretov J, Wang S (2021). THOC2 and THOC5 Regulate Stemness and Radioresistance in Triple-Negative Breast Cancer. Adv Sci (Weinh).

[8] Busato F, Khouzai BE, Mognato M (2022). Biological Mechanisms to Reduce Radioresistance and Increase the Efficacy of Radiotherapy: State of the Art. Int J Mol Sci.

[9] Lewis EB (1978). A gene complex controlling segmentation in Drosophila. Nature.

[10] Shah N, Sukumar S (2010). The Hox genes and their roles in oncogenesis. Nat Rev Cancer.

[11] Shenoy US, Adiga D, Kabekkodu SP (2022). Molecular implications of HOX genes targeting multiple signaling pathways in cancer. Cell Biol Toxicol.

[12] Boimel PJ, Cruz C, Segall JE (2011). A functional in vivo screen for regulators of tumor progression identifies HOXB2 as a regulator of tumor growth in breast cancer. Genomics.

[13] Jing P, Zou J, Zhang L (2020). HOXB2 and FOXC1 synergistically drive the progression of Wilms tumor. Exp Mol Pathol.

[14] Pan X, Liu W, Chai Y (2020). Genetic and Clinical Characterization of HOXB2 in Glioma. Onco Targets Ther.

[15] Xu F, Liu Z, Liu R (2020). Epigenetic induction of tumor stemness via the lipopolysaccharide-TET3-HOXB2 signaling axis in esophageal squamous cell carcinoma. Cell Commun Signal.

[16] Li S, Pei Y, Wang W (2019). Circular RNA 0001785 regulates the pathogenesis of osteosarcoma as a ceRNA by sponging miR-1200 to upregulate HOXB2. Cell Cycle.

[17] Shi F, Li T, Liu Z (2018). FOXO1: Another avenue for treating digestive malignancy?. Semin Cancer Biol.

[18] Wang Y, Lyu Z, Qin Y (2020). FOXO1 promotes tumor progression by increased M2 macrophage infiltration in esophageal squamous cell carcinoma. Theranostics.

[19] Bao S, Wu Q, McLendon RE (2006). Glioma stem cells promote radioresistance by preferential activation of the DNA damage response. Nature.

[20] Kelley K, Knisely J, Symons M (2016). Radioresistance of Brain Tumors. Cancers (Basel).

[21] Tolios A, De Las Rivas J, Hovig E, T (2020). Computational approaches in cancer multidrug resistance research: Identification of potential biomarkers, drug targets and drug-target interactions. Drug Resist Updat.

[22] Pan B, Zhao M, Wang N (2019). LncRNA RGMB-AS1 Promotes Glioma Growth and Invasion Through miR-1200/HOXB2 Axis. Onco Targets Ther.

[23] Yang X, Tong Y, Ye W (2022). HOXB2 increases the proliferation and invasiveness of colon cancer cells through the upregulation of CCT6A. Mol Med Rep.

[24] Zhang L, Lu Z, Zhao X (2021). Targeting Bcl-2 for cancer therapy. Biochim Biophys Acta Rev Cancer.

[25] Zhan Y, Fan S (2020). Multiple Mechanisms Involving in Radioresistance of Nasopharyngeal Carcinoma. J Cancer.

[26] Yang J, Zhu D, Liu S (2020). Curcumin enhances radiosensitization of nasopharyngeal carcinoma by regulating circRNA network. Mol Carcinog.

[27] Guo Y, Zhang Y, Zhang SJ (2019). Comprehensive analysis of key genes and microRNAs in radioresistant nasopharyngeal carcinoma. BMC Med Genomics.

[28] Zhang L, Liang B, Xu H (2022). Cinobufagin induces FOXO1-regulated apoptosis, proliferation, migration, and invasion by inhibiting G9a in non-small-cell lung cancer A549 cells. J Ethnopharmacol.

[29] Chae YC, Kim JY, Park JW (2019). FOXO1 degradation via G9a-mediated methylation promotes cell proliferation in colon cancer. Nucleic Acids Res.

[30] Wang L, Yang C, Chu M (2021). Cdc20 induces the radioresistance of bladder cancer cells by targeting FoxO1 degradation. Cancer Lett.

[31] Huang C, Chen D, Zhu H (2019). LITAF Enhances Radiosensitivity of Human Glioma Cells via the FoxO1 Pathway. Cell Mol Neurobiol.

[32] Li Y, Liu X, Lin X (2019). Chemical compound cinobufotalin potently induces FOXO1-stimulated cisplatin sensitivity by antagonizing its binding partner MYH9. Signal Transduct Target Ther.

[33] Deng L, Yin Q, Liu S (2022). MicroRNA-613 Enhances Nasopharyngeal Carcinoma Cell Radiosensitivity via the DNA Methyltransferase 3B/Tissue Inhibitor of Matrix Metalloproteinase-3/Signal Transducer and Activator of Transcription-1/Forkhead Box O-1 Axis. Dis Markers.

