# Supplementary material for: Homeobox B2 promotes malignant behavior and contributes to the radioresistance of nasopharyngeal carcinoma by regulating forkhead box protein O1
Source: Int J Med Sci. 2024 Mar 17;21(5):837–47. doi: 10.7150/ijms.93128 (PMC11008478; doi:10.7150/ijms.93128)
Supplement: Supplementary file 1 — Supplementary figure. [file ijmsv21p0837s1.pdf]

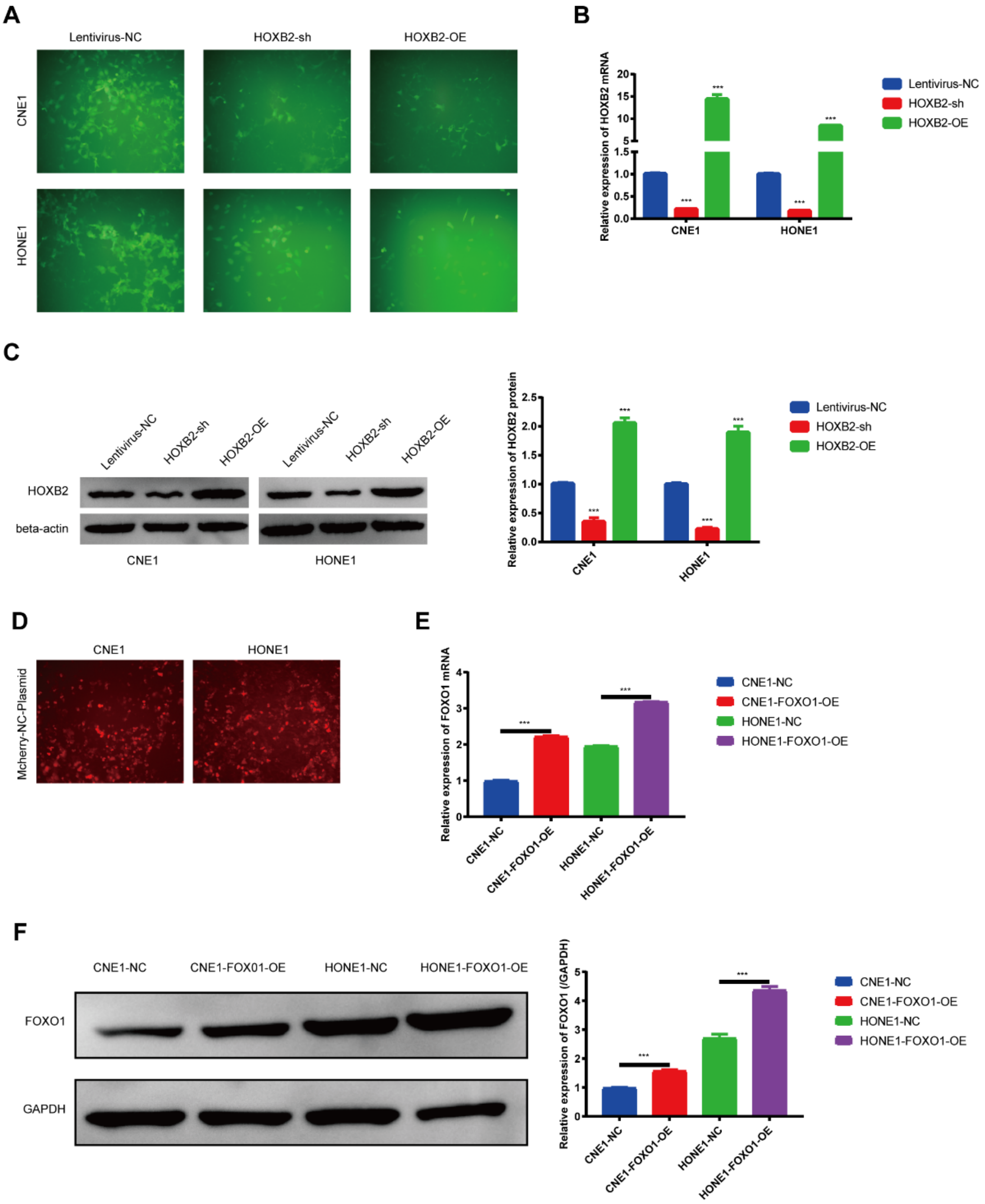

Supplementary Fig 1: Identification of Over-expression/knockdown HOXB2 or FOXO1 NPC cell lines. (A) Cell fluorescence after HOXB2 gene overexpression/knockdown constructed to NPC cells. (B) HOXB2 mRNA expression levels in constructed overexpression/knockdown NPC cells. (C) HOXB2 protein expression levels in constructed overexpression/knockdown NPC cells. (D) Mcherry-

NC-Plasmid determined the transient transfection efficiency of FOXO1 overexpression plasmid to NPC cells. (E) FOXO1 mRNA expression levels after FOXO1-overexpression-plasmid transfect to NPC cells. (F) FOXO1 protein expression levels after FOXO1-overexpression-plasmid transfect to NPC cells.

FOXO1, forkhead box protein O1; HOXB2, homeobox 2
